# Transcriptional cross talk between orphan nuclear receptor ERRγ and transmembrane transcription factor ATF6α coordinates endoplasmic reticulum stress response

**DOI:** 10.1093/nar/gkt429

**Published:** 2013-05-28

**Authors:** Jagannath Misra, Don-Kyu Kim, Woogyun Choi, Seung-Hoi Koo, Chul-Ho Lee, Sung-Hoon Back, Randal J. Kaufman, Hueng-Sik Choi

**Affiliations:** ^1^Center for Nuclear Receptor Signals, Hormone Research Center, School of Biological Science and Technology, Chonnam National University, Gwangju 500-757, Republic of Korea, ^2^School of Biological Sciences, University of Ulsan, Ulsan 680-749, South Korea, ^3^Division of Life Sciences, College of Life Sciences and Biotechnology, Korea University, 145 Anam-Ro, Seongbuk-Gu, Seoul 136-701, Republic of Korea, ^4^Korea Research Institute of Bioscience and Biotechnology, Daejeon, Republic of Korea, ^5^Center for Neuroscience, Aging, and Stem Cell Research, Sanford-Burnham Medical Research Institute, 10901 North Torrey Pines Road, La Jolla, CA 92037, USA and ^6^Research Institute of Medical Sciences, Department of Biomedical Sciences, Chonnam National University Medical School, Gwangju 501-746, Republic of Korea

## Abstract

Orphan nuclear receptor ERRγ is a member of nuclear receptor superfamily that regulates several important cellular processes including hepatic glucose and alcohol metabolism. However, mechanistic understanding of transcriptional regulation of the ERRγ gene remains to be elucidated. Here, we report that activating transcription factor 6α (ATF6α), an endoplasmic reticulum (ER)-membrane–bound basic leucine zipper (bZip) transcription factor, directly regulates ERRγ gene expression in response to ER stress. ATF6α binds to ATF6α responsive element in the ERRγ promoter. The transcriptional coactivator peroxisome proliferator-activated receptor gamma coactivator 1-α (PGC-1α) is required for this transactivation. Chromatin immunoprecipitation (ChIP) assay confirmed the binding of both ATF6α and PGC1α on the ERRγ promoter. ChIP assay demonstrated histone H3 and H4 acetylation occurs at the ATF6α and PGC1α binding site. Of interest, ERRγ along with PGC1α induce ATF6α gene transcription upon ER stress. ERRγ binds to an ERRγ responsive element in the ATF6α promoter. ChIP assay confirmed that both ERRγ and PGC1α bind to a site in the ATF6α promoter that exhibits histone H3 and H4 acetylation. Overall, for the first time our data show a novel pathway of cross talk between nuclear receptors and ER-membrane–bound transcription factors and suggest a positive feed-forward loop regulates ERRγ and ATF6α gene transcription.

## INTRODUCTION

Estrogen-related receptors (ERRs) are members of the NR3B subfamily of nuclear receptors, which include ERRα, ERRβ and ERRγ. These orphan nuclear receptors regulate transcription via estrogen response elements and the closely related ERR response elements (ERREs) but do not bind endogenous estrogen (1). The ERRs are named owing to the conservation in the structure of their DNA-binding domains with the highly homologous Estrogen Receptor (2). Crystallographic studies indicate that the ERRs along with ERRγ are constitutively active without a natural ligand, while several synthetic ligands either stimulate or repress the activity of ERRγ by promoting or disrupting ERR–coactivator interactions (3). Among them, GSK5182, a 4-hydroxy tamoxifen analog, is a selective inverse agonist of ERRγ relative to other nuclear hormone receptors (4). ERRγ is primarily expressed in heart, brain, kidney, pancreas and liver tissues (3). The transcriptional activity of the ERR family is dependent on interactions with coactivators, in particular PGC-1α and PGC-1β (5). ERRα and ERRγ regulate mitochondrial programs involved in oxidative phosphorylation and a nuclear-encoded mitochondrial genetic network that coordinates the postnatal metabolic transition in the heart (5). We previously reported that hepatic ERRγ regulates hepatic gluconeogenesis by directly binding to the Phosphoenolpyruvate carboxykinase and Glucose 6-phosphatase (G6Pase) promoters along with coactivator PGC-1α (6). Previous results from our laboratory also demonstrated that ERRγ directly binds to the LIPIN1 promoter along with coactivator PGC-1α to regulate LIPIN1 gene expression, and inhibits hepatic insulin signaling (7), ERRγ controls hepatic CB1 receptor-mediated CYP2E1 expression at the transcriptional level and thus contributes to the oxidative liver injury by alcohol (8). Finally, hypoxia induces pyruvate dehydrogenase kinase 4 (PDK4) gene expression through induction of ERRγ (9). Although all these reports clearly suggest a key role of ERRγ in different cellular processes, its role in endoplasmic reticulum (ER) stress is yet to be determined.

Recently, numerous studies demonstrate the importance of ER stress in the pathogenesis of various liver diseases, including chronic viral hepatitis, insulin resistance, nonalcoholic fatty liver disease, ischemia-reperfusion injury, genetic disorders of protein misfolding and alcoholic liver disease (10–12). The ER stress response involves the function of three molecular components: protein kinase R-like ER kinase, inositol requiring enzyme-1/X-box binding protein (XBP)-1 and activating transcription factor 6α (ATF6α) (13). Among these, ATF6α is a member of the ATF/cAMP response element-binding protein basic-leucine zipper family of DNA-binding proteins (14). On induction of ER stress, ATF6α translocates from the ER to the Golgi (15), where it is cleaved by site 1 and 2 proteases (16). Proteolytic cleavage of ATF6α directly induces transcriptional activation of ER chaperones and other enzymes that are essential for protein folding (15–18). In addition to posttranslational modification of ATF6α, accumulating evidence suggests that ER stressors, including hypoxia and tunicamycin (Tm) upregulate ATF6α mRNA expression, which suggests that an increase in the expression of ATF6α is also important for the ER stress response (16,19). ATF6α has been reported to regulate Glucose-Regulated Protein78 (GRP78) gene expression (20). ATF6α also interacts with serum response factor to regulate serum-induced expression of the c-fos gene (21). One report also suggests that ATF6α concertedly works with coactivator PGC1α to regulate different gene expression (22). Although all these reports clearly suggest a key role of ATF6α in regulation of different cellular factors, its role in regulation of nuclear receptors during ER stress is yet to be determined.

PGC-1α, a member of a small family of coactivators, was identified using yeast two-hybrid assays for peroxisome proliferator-activated receptor γ (PPARγ)-interacting proteins (23) and is implicated in mitochondrial metabolism, thermogenesis, mitochondrial biogenesis, adipocyte differentiation, gluconeogenesis and glucose uptake (24,25) and to interact with a number of other nuclear receptors such as glucocorticoid receptor (GR) (26), nuclear respiratory factor-1 (NRF-1) (27), hepatocyte nuclear factor 4α (HNF4α) (28), estrogen receptor α (ERα) (29), peroxisome proliferator-activated receptor α (PPARα) (30), retinoid X receptor (RXR) (31) and ERRα (32). PGC-1α is a 798 amino acid multifunctional protein harboring several domains with distinct activities. The amino-terminal domain exhibits a transcriptional activation function (29) that is followed by an overlapping region involved in interactions with nuclear receptors containing two well-characterized NR boxes required for receptor recognition (24,29,31,33). For ligand-dependent interaction with the nuclear receptors at least one of the three NR box motifs of PGC-1α is required (29,31,33). The lysine-rich region (residues 214–250) is thought to contain putative nuclear localization signals. PGC-1α targets promoters by interacting directly with numerous DNA-binding transcription factors and then coordinating several biochemical events, including recruitment of chromatin-modifying enzymes such as p300/CREB-binding protein (CBP) and steroid receptor coactivator-1 (SRC-1), interaction with the basal transcription machinery and linking of transcription to RNA splicing (34).

Previous reports suggest different nuclear receptors and many transcription factors cross talk to regulate mammalian gene expression in response to ER stress. ER stress–induced activation of ATF6α decreases insulin gene expression via upregulation of orphan nuclear receptor small heterodimer partner (SHP) (35). One report suggests that nuclear receptor PPARβ/δ is regulated by ATF4 (36). XBP1 increases ERα transcriptional activity (37,38). ER-membrane–bound cyclic AMP responsive element binding protein-H (CREBH) is regulated by ER stress (39), fatty acids and PPARα (40) and HNF4α (41). Several reports also demonstrate that PPARδ activation rescues pancreatic β-cells from palmitate-induced ER stress through enhanced fatty acid oxidation (42). Furthermore, ER stress–induced CHOP (C/EBP Homologous Protein) enhances nuclear factor-κβ (NF-κβ) signaling via repression of PPARγ (43). Therefore, all these previous reports indicate that different nuclear receptors and transcription factors coordinate the mammalian ER stress response under different physiological conditions.

Here, we examined the mechanism of cross talk between a nuclear receptor, ERRγ and an ER-membrane–bound bZIP transcription factor, ATF6α. In response to ER stress, expression of both transcription factors increases significantly through reciprocal activation. ATF6α directly binds to the ERRγ promoter, and ERRγ directly binds to the ATF6α promoter. Of most interest, PGC1α acts as a coactivator in both cases. The physical interaction of PGC1α with both ERRγ and ATF6α increases significantly upon ER stress that identifies the importance of PGC1α in this cross talk. We observed a significant increase in histone H3 and H4 acetylation in both promoters upon induction of ER stress by Tm treatment. Knockdown of either factor significantly decreases the expression of the other, suggesting their ability to *trans*-activate each other. Moreover, our study reveals ERRγ is induced earlier that ATF6α in response to ER stress. Together, we present a novel mechanistic pathway that would encourage further study to elucidate the relation between nuclear receptors and ER stress.

## MATERIALS AND METHODS

### Chemicals and antibodies

Tm and Brefeldin A were obtained from Sigma-Aldrich; Thapsigargin was obtained from Sigma, and GSK5182 was synthesized as described previously (6). Antibodies used in this work were as follows: anti-ERRγ (Perseus Proteomics), anti-ERRα (abcam), anti-tubulin (Ab_FRONTIER_), anti-ATF6 (Imgenex), anti-PGC1α (Santa Cruz), anti-acetyl-histone H3 (Cell Signaling), anti-acetyl-histone H4 (Cell Signaling), anti-GRP78 (abcam), anti-PDK4 (abcam), anti-CHOP (Santa cruz), anti-XBP1 (Santa cruz) and anti-ATF4 (Santa cruz). The primary antibodies were used at a dilution ranging from 1:200 to 1:1000 for western blot analysis, and at a dilution of 1:200 for immunoprecipitation.

### Plasmids and adenovirus

The reporter plasmids mERRγ-Luc (6), hPDK4-Luc, ERRγ responsive element (ERRRE) mutant PDK4-Luc (9), GRP78-Luc (44) were described previously. Expression vector for FLAG-ERRγ (7), PGC1α (6), ATF4 (45) and ATF6α (17) were described previously. The coding sequences for XBP-1 and CHOP were amplified from mouse hepatic cDNAs and inserted into pcDNA3-flag to generate pcDNA3-flag-XBP-1 and pcDNA3-flag-CHOP. ATF6α responsive element (ATF6αRE) mutant GRP78 promoter was made using wild-type GRP78 promoter (−0.457 kb) as template by Quick Change Lightning Site-Directed Mutagenesis kit from Agilent Technologies. All mouse ERRγ promoters were cloned from mouse genomic DNA and inserted into pGL3-Basic vector using Mlu1/Xho1 restriction sites. Mutant ERRγ promoter was made using wild-type ERRγ promoter (−1.5 kb) as template by Quick Change Lightning Site-Directed Mutagenesis kit from Agilent Technologies. All mouse ATF6α promoters were cloned from mouse genomic DNA and inserted into pGL3-Basic vector using Mlu1/Xho1 restriction sites. Mutant ATF6α promoter was made using wild-type ATF6α promoter (−2.6 kb) as template by Quick Change Lightning Site-Directed Mutagenesis kit from Agilent Technologies. All primer sequences are listed in supplemental table. All plasmids were confirmed via DNA sequence analysis. For ectopic expression of the genes, adenoviral delivery was used. Adenoviruses (Ad) encoding GFP only (Ad–GFP), Ad–ERRγ, Ad–shERRγ, Ad–ATF6α, Ad–PGC1α and Ad–shPGC1α were described elsewhere (6,7,46,47). Adenovirus-encoding shATF6α was prepared as follows. Briefly, the shATF6α (AGAGAAGCCTGTCACTGGTCCTGGAAA) constructs were made with a 27-mer double-stranded oligonucleotide spanning +411 to +437 of the ATF6α cDNA sequence in the pBS/U6 vector. The cDNA-encoding shATF6α was cloned into the pAdTrack-CMV vector. The recombination of the pAdTrack-CMV-shATF6α with adenoviral gene carrier vector was performed by transformation using adEasy-BJ21-competent cells. The primers used for shATF6α construction are as follows: h/mATF6α forward 5′- AGAGAAGCCTGTCACTGGTCCTGGAAAaagcttTTTCCAGGACCAGTGACAGGCTTCTCTctttttgc-3′ and reverse 5′-ggccgcaaaaagAGAGAAGCCTGTCACTGGTCCTGGAAAaagcttTTTCCAGGACCAGTGACAGGCTTCTCT-3′.

### Cell culture, transient transfection and luciferase assay

AML12, 293T and HeLa cells were obtained from the American Type Culture Collection. Maintenance of cell lines and transient transfection assays were performed using Lipofectamine2000 transfection reagent (Invitrogen) according the manufacturer’s instructions as described elsewhere (48). Briefly, cells were transfected with indicated reporter plasmids together with expression vectors encoding various transcription factors or treated with various chemicals. Total cDNA used for each transfection was adjusted to 1 µg/well by adding appropriate amount of empty vector and pCMV–β-gal plasmid was used as an internal control. The luciferase activity was normalized to β-galactosidase activity and expressed as relative luciferase units. The generation of ATF6α-null hepatocyte cell lines from ATF6α-null mice was previously described (18,49).

### Coimmunoprecipitation assay and western blot analysis

Coimmunoprecipitation (Co-IP) and western blot analyses were performed as described previously (48). For Co-IP from tissue extracts, C57BL/6J mice (*n* = 5) were maintained *ad libitum* for desired experimental period and sacrificed. Liver tissue samples were used for Co-IP assay. For western blot analysis, cell lysates were prepared and analyzed as previously described (50).

### Confocal microscopy

Confocal microscopy was performed as described elsewhere (48). In brief, the HeLa cells grown on gelatin-coated coverslips were transfected using Lipofectamine2000 transfection reagent (Invitrogen) according to the manufacturer’s instructions. At 24 h after transfection, the cells were fixed with 2% formaldehyde, immunostained and subjected to observation by confocal microscopy.

### RNA interference

Knockdown of PGC1α was performed using the pSuper vector system (50,51). AML12 cells were transfected with siRNA constructs using Lipofectamine 2000 (Invitrogen) according to the manufacturer’s instructions. siRNA-treated cells were analyzed using reverse transcription polymerase chain reaction (RT-PCR) to measure the extent of knockdown.

### Reverse transcriptase PCR and quantitative real-time PCR analysis

Total RNA was isolated using the TRIzol reagent (Invitrogen) according to the manufacturer’s instructions. The mRNAs of ATF6α and PGC1α were analyzed by RT-PCR or quantitative real-time RT-PCR (qPCR) as indicated. DNA samples from total RNA reverse transcription or from chromatin immunoprecipitation (ChIP) assays served as the templates for qPCR, which were performed with TOPreal SYBR Green PCR Kit (Enzynomics) and the Step One Plus real-time PCR system (Applied Bioscience) in triplicate. mRNA expression levels were normalized to those of β-actin (ACTB). The RT–PCR and qPCR primer sequences are available on request.

### ChIP assay

Formaldehyde cross-linking of cells, ChIPs and real-time PCR analyses were performed as described elsewhere (5). After sonication, soluble chromatin was subjected to immunoprecipitation using anti-ATF6α, anti-PGC1α, anti-Acetyl Histone 3, anti-Acetyl Histone 4 and anti-ERRγ antibody. DNA was recovered by phenol/chloroform extraction and analyzed by PCR and/or qPCR using primers against relevant promoters.

### Animal experiments

Male 7–12-week-old C57BL/6J mice (Charles River Laboratories) were maintained on a 12-h/12-h light/dark cycle and fed *ad libitum*. Tm (1 mg/kg, i.p., in 1% DMSO/DW) was administered by Intraperitoneal injection into C57BL/6J mice (*n* = 5 per group) for 0–12 h for time-course study, or Ad-GFP or Ad-ERRγ were injected via tail vein into male C57BL/6J mice (*n* = 5 per group). Where indicated, GSK5182 was administered first (40 mg/kg, p.o., in 30% PEG400/DW) by intraperitoneal injection, and after 30 min, Tm (1 mg/kg, i.p., in 1% DMSO/DW) was administered by intraperitoneal injection into C57BL/6J mice (*n* = 5 per group). All experiments were conducted as previously described (7) under the guidelines of the Korea Research Institute of Bioscience and Biotechnology Animal Care and Use Committee.

### Statistical analysis

Data are expressed as means ± SEM. Statistical analysis was performed using the two-tailed Student *t* test. Differences were considered statistically significant at *P* < 0.05.

## RESULTS

### ER stress induces ERRγ gene expression

**Figure 1.**
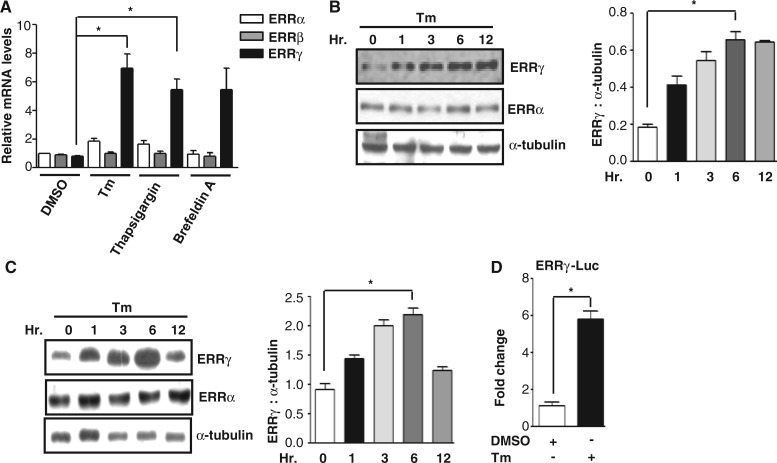
ER stress induces ERRγ gene expression. (**A**) AML12 cells were treated with Tm (5 µg/ml), Thapsigargin (0.5 µmol/L) and Brefeldin A (30 ng/ml) for 12 h. Total RNA was isolated from cells to perform qRT-PCR to quantify ERRα, ERRβ and ERRγ mRNA level using ERRα, ERRβ and ERRγ primers. Data are representative of three independently performed experiments and shown as mean ± SD; *P < 0.05 using Student’s *t*-test. (**B**) AML12 cells were treated with Tm (5 µg/ml) for increasing periods of time up to 12 h. Western blot analysis shows ERRγ expression. Quantification of immunoblot is also shown. *P < 0.05 using Student’s *t*-test. Data are representative of three independently performed experiments. (**C**) Tm (1 mg/kg, i.p., in 1% DMSO/DW) was administered into C57BL/6J mice (*n* = 5 per group) for up to 12 h. Following completion of the experiments, mice were sacrificed, and liver tissues were obtained for western blot analyses of ERRγ. Quantification of immunoblot is also shown. *P < 0.05 using Student’s *t*-test. (**D**) Tm-dependent activation of the ERRγ promoter. 293T cells were transfected with ERRγ-Luc (200 ng). At 24 h after transfection, cells were serum starved for 24 h, followed by DMSO or Tm treatment (5 µg/ml) for 12 h. Data are representative of three independently performed experiments and shown as mean ± SD; *P < 0.05 using Student’s *t*-test.

ER stress promotes LIPIN2-dependent hepatic insulin resistance (45) and ERRγ is a novel transcriptional regulator of LIPIN1, and inhibits hepatic insulin signaling (7). To investigate whether there was any connection between ER stress and ERRγ, AML12 (Mouse hepatoma cell line) cells were treated with known ER stress inducers, Tm, Thapsigargin (45) and Brefeldin A (52). A significant increase in the ERRγ mRNA level was observed by qRT-PCR, though there was no significant increase in ERRα and ERRβ mRNA level (Figure 1A). Similar results were obtained when western blot analysis was performed after treating AML12 cells with Tm in a time-dependent manner (Figure 1B). To test the effect of Tm *in vivo*, mice were injected with Tm. A significant increase in ERRγ mRNA (Supplementary Figure S1A) and protein (Figure 1C) was observed in a time-dependent manner reconfirming our *in vitro* findings. To evaluate the potential role of ER stress on ERRγ promoter activity, a transient transfection assay was performed with an ERRγ promoter containing reporter. Tm treatment significantly increased ERRγ promoter activity (Figure 1D). Taken together, these initial results indicate that ER stress specifically increases ERRγ gene expression in hepatocytes.

### ER stress induces ERRγ gene expression via ATF6α

**Figure 2.**
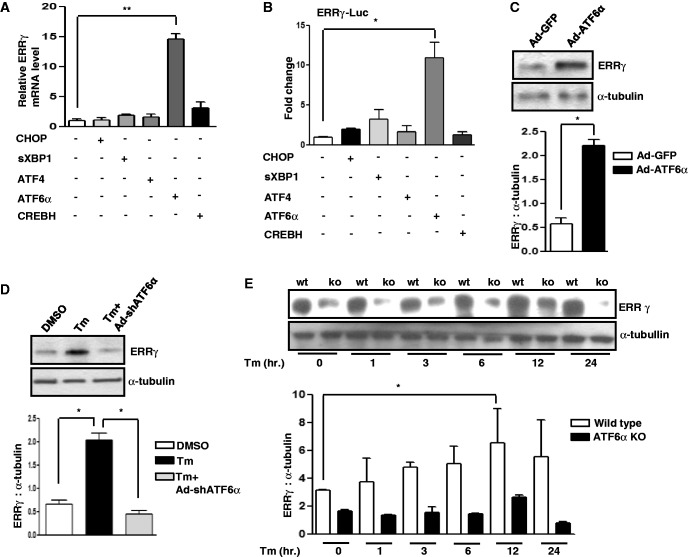
ER stress induces ERRγ gene expression via ATF6α. (**A**) AML12 cells were transfected with CHOP, XBP1, ATF4, ATF6α and CREBH plasmid DNAs. Total RNA was isolated for qRT-PCR analysis to quantify ERRγ mRNA level using ERRγ primers. Data are representative of three independently performed experiments and shown as mean ± SD; **P < 0.005 using Student’s *t*-test. (**B**) ATF6α-dependent activation of the ERRγ promoter. Transient transfection was performed in 293T cells with the indicated plasmid DNAs. Data are representative of three independently performed experiments and shown as mean ± SD; *P < 0.05 using Student’s *t*-test. (**C**) AML12 cells were infected with Ad-GFP or Ad-ATF6α for 24 h. Western blot analysis shows expression of ERRγ. Quantification of immunoblot is also shown. *P < 0.05 using Student’s *t*-test. Data are representative of three independently performed experiments. (**D**) AML12 cells were treated with DMSO or Tm or first infected with Ad-shATF6α, and at 48 h after infection, treated with Tm (5 µg/ml) for 12 h. Western blot analysis shows ERRγ expression. Quantification of immunoblot is also shown. *P < 0.05 using Student’s *t*-test. Data are representative of three independently performed experiments. (**E**) Atf6α-null hepatocyte cell lines were treated with Tm for increasing times up to 24 h. Western blot analysis shows ERRγ expression. Quantification of immunoblot is also shown. *P < 0.05 using Student’s *t*-test. Data are representative of three independently performed experiments.

The response to ER stress is a coordination of signaling pathways that activates several transcription factors including ATF6α, CREBH, ATF4, XBP1, and CHOP (53). These transcription factors induce different genes involved in numerous biological processes (6,7,44,54,55). To assess whether any of these transcription factors was involved in ER stress mediated induction of ERRγ gene expression, XBP1, ATF4, ATF6α, CHOP or CREBH cDNAs were overexpressed in AML12 cells. Interestingly, only ATF6α overexpression significantly increased ERRγ mRNA level (Figure 2A). To further elucidate the role of these factors, transient transfection assays were performed with the ERRγ promoter containing reporter along with XBP1, ATF4, ATF6α, CHOP or CREBH expression vectors. Only ATF6α significantly (>10 fold) activated the ERRγ promoter, consistent with our previous observation (Figure 2B). Though the promoter activity was also increased in the presence of XBP1, the increase was negligible compared to ATF6α. To confirm the role of ATF6α, next we checked the effect of adenovirus mediated overexpression of ATF6α (Ad-ATF6α) in AML12 cells. More than 4 fold increase in ERRγ protein level was observed in presence of Ad-ATF6α compared to control (Figure 2C). Similar results were obtained when ATF6α was overexpressed in HepG2 cells (human hepatoma cell line) (Supplementary Figure S1B and S1C). All these results indicated that ER stress induced ERRγ gene expression was mediated through ATF6α. To test this hypothesis, endogenous ATF6α was knocked down by Ad-shATF6α in presence of Tm in AML12 cells. As expected, the Tm mediated increase in ERRγ protein level was significantly decreased in response to ATF6α knock down (Figure 2D). To further verify the role of ATF6α, wild-type and Atf6α-deleted immortalized hepatocyte cell lines were treated with Tm. The ERRγ protein level (Figure 2E) was significantly reduced in Atf6α-deleted cells compared to wild-type cells under basal conditions, as well as after Tm treatment. Collectively these results demonstrate that ATF6α mediates the induction of ERRγ gene expression by ER stress.

### ATF6α induces ERRγ gene transcription via an ATF6αRE

**Figure 3.**
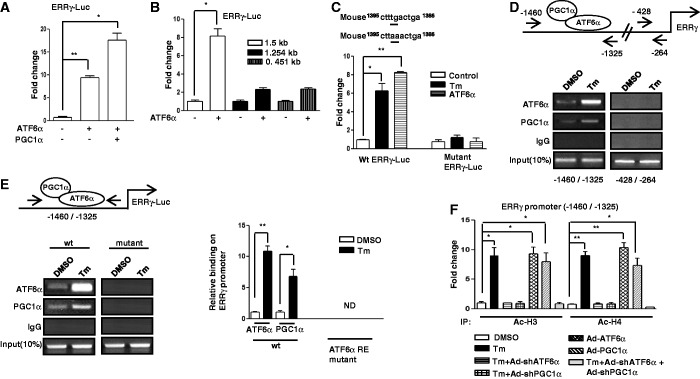
ATF6α regulates ERRγ via ATF6αRE. (**A**) PGC1α-dependent activation of the ERRγ promoter by ATF6α. Transient transfection was performed in 293T cells with the indicated plasmid DNAs. Data are representative of three independently performed experiments and shown as mean ± SD; *P < 0.05 and **P < 0.005 using Student’s *t*-test. (**B**) Deletion constructs of the ERRγ promoter show the ATF6α binding site in 293T cells. Transient transfection was performed in 293T cells with the indicated plasmid DNAs. Data are representative of three independently performed experiments and shown as mean ± SD; *P < 0.05 using Student’s *t*-test. (**C**) ATF6αRE-dependent activation of the ERRγ promoter in 293T cells. 293T cells were transfected with wild-type or ATF6αRE-mutant ERRγ promoter along with ATF6α plasmid DNAs or treated with Tm (5 µg/ml). Data are representative of three independently performed experiments and shown as mean ± SD; *P < 0.05 and **P < 0.005 using Student’s *t*-test. (**D**) ChIP assay to detect the binding of ATF6α and PGC1α to the endogenous ERRγ promoter by semiquantitative PCR. AML12 cells were treated with DMSO or Tm for 12 h. After completion of the treatment, Chromatin fragments were prepared and immunoprecipitated with ATF6α, PGC1α or IgG control antibodies. DNA fragments covering −1460 to −1325 and −428 to −264 elements on the ERRγ promoter were PCR-amplified. Ten percent of the soluble chromatin was used as input. Data are representative of three individually performed experiments. (**E**) AML12 cells were transfected with wild-type or TGAC-mutant ERRγ promoter. Following transfection, cells were treated with DMSO or Tm (5 µg/ml) for 12 h. Soluble chromatin was prepared and immunoprecipitated with antibody against ATF6α, PGC1α or IgG only as indicated. Ten percent of the soluble chromatin was used as input. Semiquantitative PCR (left panel) and qPCR (right panel) was performed to determine and quantify the binding of ATF6α and PGC1α to transfected ERRγ promoter. Data are representative of three individually performed experiments. *P < 0.05 and **P < 0.005 using Student’s *t*-test. ND, not detectable. (**F**) ChIP assay for detection of histone acetylation at the ATF6α/PGC1α binding site in the endogenous ERRγ promoter under the indicated conditions in AML12 cells. Chromatin fragments were prepared and immunoprecipitated with Acetyl-Histone 3 and Acetyl-Histone 4 antibodies. DNA fragments covering −1460 to −1325 elements in the ERRγ promoter were qPCR-amplified as described in the ‘Materials and Methods’ section. Data are representative of three independently performed experiments and shown as mean ± SD; *P < 0.05 and **P < 0.005 using Student’s *t*-test.

Next, we attempted to ascertain the molecular mechanism of ATF6α-mediated ERRγ gene induction. It was previously reported that PGC1α acts as a coactivator of ATF6α (22). To test whether PGC1α along with ATF6α has any role in the induction of ERRγ, transient transfection assays were performed with mouse ERRγ promoter containing reporter and ATF6α and PGC1α expression vectors. ATF6α significantly increased ERRγ promoter activity, and this activity was further increased in the presence of PGC1α (>15-fold) (Figure 3A). Similar results were obtained for the human ERRγ promoter (Supplementary Figure S1D). To identify the DNA sequence conferring ATF6α-mediated ER stress effect on the ERRγ promoter, a series of deletion constructs was analyzed. Deletion of the ERRγ promoter sequence from 1.5 to 1.253 kb drastically decreased the promoter activity conferred by ATF6α, suggesting that the region from 1.5 to 1.253 bp conferred the activation of ERRγ promoter (Figure 3B). It was previously reported that ATF6α binds to a consensus sequence (G)(G)TGACGTG(G/A) (17). We aligned this sequence with ERRγ promoter sequence, and although we could not find a perfect match to the consensus sequence, TTTGACTGAG spanning region 1.5–1.253 kb was found. To test whether TGAC may be the core sequence critical for ATF6α binding, transient transfection assays were performed using wild-type and TGAC-mutant reporters with Tm and ATF6α. This mutant reporter did not show any significant response to either Tm treatment or ATF6α cotransfection (Figure 3C). Next, ChIP assay was performed to monitor the effect of Tm on ATF6α and PGC1α recruitment to the endogenous ERRγ gene promoter. Under basal conditions, both ATF6α and PGC1α occupied the ERRγ promoter. However, Tm treatment significantly augmented ATF6α and PGC1α occupancy on the ERRγ promoter (Figure 3D). To further confirm the binding site, ChIP assay was performed with the wild-type and TGAC-mutant ERRγ promoter. The results demonstrated that ATF6α and PGC1α were present in ERRγ promoter and Tm further induced ATF6α and PGC1α binding to ERRγ chromatin. As expected, no binding was observed in the TGAC mutant ERRγ promoter (Figure 3E), therefore suggesting that Tm treatment activates the ERRγ gene transcription via enhancing ATF6α and PGC1α binding to the promoter. The ChIP assay results provide critical *in vivo* evidence that the Tm/ATF6α-PGC1α signaling pathway increases ERRγ gene transcription. Because gene activation is often associated with increased histone acetylation (57), to determine whether Tm treatment results in increased template-associated histone (H3 and/or H4) acetylation of the ERRγ gene promoter, ChIP assay was performed (Figure 3F). Tm treatment as well as adenoviral overexpression of ATF6α or PGC1α increased acetylation of H3 (Ac–H3) and H4 (Ac–H4) on the ATF6α-responsive region of ERRγ promoter, whereas knockdown of endogenous ATF6α or endogenous PGC1α significantly reduced the histone (H3 and/or H4) acetylation. Overall these results demonstrate that ER stress augments the binding of ATF6α and PGC1α to the ERRγ promoter and increases template-associated histone (H3 and H4) acetylation to facilitate ERRγ gene transcription.

### ER stress induces ATF6α gene expression via ERRγ

**Figure 4.**
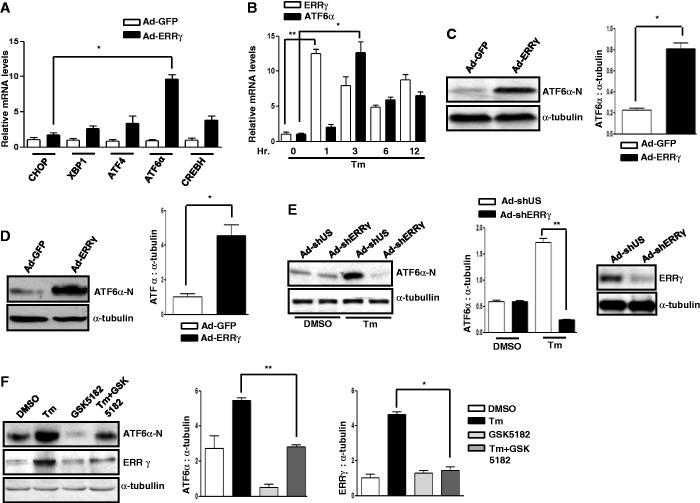
ATF6α and ERRγ cross talk in gene regulation. (**A**) AML12 cells were infected with Ad-GFP or Ad-ERRγ for 24 h. Total RNA was isolated for qRT-PCR analysis to quantify CHOP, XBP1, ATF4, ATF6α and CREBH mRNA levels using CHOP, XBP1, ATF4, ATF6α and CREBH primers. Data are representative of three independently performed experiments and shown as mean ± SD; *P < 0.05 using Student’s *t*-test. (**B**) AML12 cells were treated with Tm (5 µg/ml) for increasing periods of time up to 12 h. Total RNA was isolated for qRT-PCR analysis to quantify ERRγ and ATF6 mRNA levels using ERRγ and ATF6α primers. Data are representative of three independently performed experiments and shown as mean ± SD; *P < 0.05, and **P < 0.005 using Student’s *t*-test. (**C**) AML12 cells were infected with Ad-GFP or Ad-ERRγ for 24 h. Western blot analysis shows expression of ATF6α-N. Quantification of immunoblot is also shown. *P < 0.05 using Student’s *t*-test. Data are representative of three independently performed experiments. (**D**) Ad-GFP or Ad-ERRγ were injected via tail vein into male C57BL/6J mice (*n* = 5 per group). Following completion of the experiments, mice were sacrificed, and liver tissues were obtained for western blot analyses of ATF6α-N. Quantification of immunoblot is also shown. *P < 0.05 using Student’s *t*-test. (**E**) AML12 cells were infected with Ad-shUS or Ad-shERRγ. At 48 h after infection, cells were treated with DMSO or Tm (5 µg/ml) for 12 h. Western blot analysis (left panel) shows ATF6α-N expression and (right panel) shows ERRγ expression. Quantification of immunoblot is also shown. **P < 0.005 using Student’s *t*-test. Data are representative of three independently performed experiments. (**F**) GSK5182 (40 mg/kg, p.o., in 30% PEG400/DW) was administered before and at 30 min after Tm injection (1 mg/kg, i.p., in 1% DMSO/DW) was administered into C57BL/6J mice (*n* = 5 per group). Following completion of the experiments, mice were sacrificed, and liver tissues were obtained for western blot analyses of ATF6α-N and ERRγ. Quantification of immunoblot is also shown. *P < 0.05, and **P < 0.005 using Student’s *t*-test.

Previous reports suggest different nuclear receptors and transcription factors regulating mammalian ER stress response cross talk with each other under conditions of ER (38,40,43,56). In addition, the nuclear receptor HNF4α regulates transcription of CREBH (38). To investigate whether ERRγ regulates any transcription factors regulating mammalian ER stress response, ERRγ was overexpressed by adenovirus (Ad-ERRγ) in AML12 cells. Surprisingly, a >10-fold increase in ATF6α mRNA level was observed, and although XBP1, ATF4 and CREBH mRNA levels also increased to some extent, the increase was insignificant compared with ATF6α (Figure 4A). Similar results were obtained when ERRγ was overexpressed in HepG2 cells (Supplementary Figure S3A). Because Tm induces transcription of ATF6α (16) and ERRγ (Figure 1), we examined the time-course of ERRγ and ATF6α induction in response to ER stress in AML12 cells. Tm induced expression of ERRγ within 1 h, whereas ATF6α optimal expression took almost 3 h. Thus, ERRγ is apparently induced before ATF6α in response to ER stress (Figure 4B). Furthermore, overexpression of ERRγ in AML12 cells (Figure 4C) and in mouse liver tissue (Figure 4D) resulted in almost 4- and 4.5-fold increase in ATF6α active form (ATF6α-N), respectively. Similar results were obtained when ERRγ was overexpressed in HepG2 cells (Supplementary Figure S3B). As we noticed an early gene induction of ERRγ compared with ATF6α upon ER stress (Figure 4B) and overexpression of ERRγ increased ATF6α expression both *in vivo* and *in vitro* (Figure 4A, C and D), we speculated that ERRγ could be responsible for the Tm-mediated increase in ATF6α gene expression. To verify this, we knocked down endogenous ERRγ by Ad-shERRγ in AML12 cells. As expected, the Tm-mediated increase in ATF6α-N protein level was significantly reduced after ERRγ knockdown (Figure 4E). Similar results were observed for ATF6α mRNA level (Supplementary Figure S3C). To further confirm this, GSK5182, an inverse agonist of ERRγ (6), which specifically binds to ERRγ and inhibits transcriptional activity of ERRγ, was used. In agreement with the previous results, we noticed that GSK5182 treatment substantially reduced the protein level of ATF6α-N in mouse liver (Figure 4F). Overall we demonstrate that during ER stress, ATF6α mediates the increase in ERRγ gene expression, ERRγ in turn mediates the increase in ATF6α gene expression.

### ERRγ regulates ATF6α transcription via an ERRE

**Figure 5.**
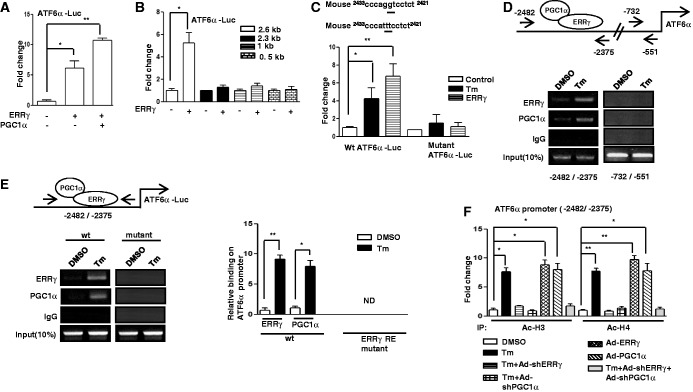
ERRγ controls transcription of ATF6α. (**A**) PGC1α-dependent activation of the ATF6α promoter by ERRγ. Transient transfection was performed in 293T cells with the indicated plasmid DNAs. Data are representative of three independently performed experiments and shown as mean ± SD; *P < 0.05 and **P < 0.005 using Student’s *t*-test. (**B**) Deletion constructs of the ATF6α promoter demonstrate the ERRγ binding site in 293T cells. Transient transfection was performed in 293T cells with the indicated plasmid DNAs. Data are representative of three independently performed experiments and shown as mean ± SD; *P < 0.05 using Student’s *t*-test. (**C**) ERRRE-dependent activation of the ATF6α promoter in 293T cells. 293T cells were transfected with wild-type or ERRRE-mutant ATF6α promoter along with ERRγ plasmid DNAs or treated with Tm (5 µg/ml). Data are representative of three independently performed experiments and shown as mean ± SD; *P < 0.05 and **P < 0.005 using Student’s *t*-test. (**D**) ChIP assay shows the binding of ERRγ and PGC1α to the endogenous ATF6α promoter by semiquantitative PCR. AML12 cells were treated with DMSO or Tm for 12 h. After completion of the treatment, chromatin fragments were prepared and immunoprecipitated with ERRγ, PGC1α or IgG control antibodies. DNA fragments covering −2482 to −2375 and −732 to −551 elements on the ERRγ promoter were PCR-amplified. Ten percent of the soluble chromatin was used as input. Data are representative of three individually performed experiments. (**E**) AML12 cells were transfected with wild-type or AGGTCC-mutant ATF6α promoter. Following transfection, cells were treated with DMSO or Tm (5 µg/ml) for 12 h. Soluble chromatin was prepared and immunoprecipitated with antibody against ERRγ, PGC1α or IgG only as indicated. Ten percent of the soluble chromatin was used as input. Semiquantitative PCR (left panel) and qPCR (right panel) were performed to determine and quantify the binding of ERRγ and PGC1α to transfected ATF6α promoter. Data are representative of three individually performed experiments. *P < 0.05 and **P < 0.005 using Student’s *t*-test. ND, not detectable. (**F**) ChIP assay for detection of histone acetylation at the ERRγ/PGC1α binding site under the indicated conditions in AML12 cells. Chromatin fragments were prepared and immunoprecipitated with Acetyl-Histone 3 and Acetyl-Histone 4 antibodies. DNA fragments covering −2482 to −2375 element on ATF6α promoter were qPCR-amplified as described in the ‘Materials and Methods’ section. Data are representative of three independently performed experiments and shown as mean ± SD; *P < 0.05 and **P < 0.005 using Student’s *t*-test.

Next, we focused on the molecular mechanism of how ERRγ overexpression led to the increase in ATF6α protein. A transient transfection assay was performed with the ATF6α promoter along with ERRγ and PGC1α expression vectors, as PGC1α acts as a coactivator of ERRγ (6). ERRγ significantly increased ATF6α promoter activity, and this was further augmented in presence of PGC1α (Figure 5A). Similar results were obtained for the human ATF6α promoter (Supplementary Figure S3D). To identify the DNA sequence conferring ERRγ-mediated ER stress effect on the ATF6α promoter, we used a series of deletion constructs of ATF6α promoter for transient transfection assay. Deletion of the ATF6α promoter sequence from 2.6 to 2.3 kb drastically decreased the promoter activity conferred by ERRγ, suggesting that the region from 2.6 to 2.3 kb conferred the activation of the ATF6α promoter (Figure 5B). It was previously reported that ERRγ binds to a sequence AGGTCA (7). A close investigation of the ATF6α promoter revealed a close sequence, AGGTCC, in between region 2.6 kb and 2.3 kb. To check whether the sequence AGGTCC mediates Tm- or ERRγ-induced activation of ATF6α, transient transfection assays were performed using wild-type and AGGTCC-mutant reporters with Tm treatment or ERRγ expression vector. This mutant reporter did not respond significantly to either Tm treatment or ERRγ coexpression (Figure 5C). Next we performed ChIP assay to detect whether endogenous ERRγ or PGC1α binds to the ATF6α promoter upon Tm treatment. Tm treatment significantly increased ERRγ and PGC1α occupancy to the ATF6α promoter compared with control cells (Figure 5D). To further confirm the binding site, ChIP assay was performed with the wild-type and mutant ATF6α promoter. ChIP assay results demonstrated that ERRγ and PGC1α were present in ATF6α promoter, and Tm treatment further induced ERRγ and PGC1α binding to the ATF6α promoter. We could not detect any binding for AGGTCC mutant ATF6α promoters (Figure 5E), indicating that Tm activates the ATF6α gene transcription via enhancing ERRγ and PGC1α binding to the promoter. We also observed a significant increase in acetylation of H3 (Ac–H3) and H4 (Ac–H4) on ATF6α promoter in response to Tm treatment as well as adenoviral overexpression of ERRγ or PGC1α, whereas knockdown of endogenous ERRγ or PGC1α resulted in significant decrease in acetylation of histone even after Tm treatment (Figure 5F). As a whole, these results describe that ERRγ mediates induction of the ATF6α gene on ER stress.

### PGC1α regulates transcriptional cross talk of ATF6α and ERRγ

**Figure 6.**
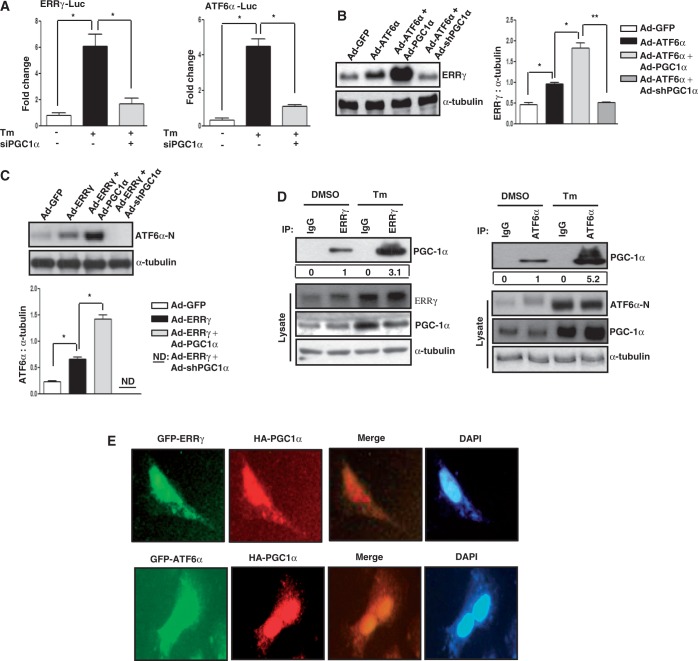
PGC1α regulates ATF6α–ERRγ cross talk. (**A**) Activation of the ERRγ promoter (left panel) and the ATF6α promoter (right panel) by Tm is mediated through PGC1α. Transient transfection was performed in AML12 cells with the indicated plasmid DNAs followed by Tm (5 µg/ml) treatment for 12 h. Data are representative of three independently performed experiments and shown as mean ± SD; *P < 0.05 using Student’s *t*-test. (**B**) AML12 cells were infected with Ad-GFP, Ad-ATF6α or Ad-ATF6α along with Ad-PGC1α for 24 h, or first infected with Ad-shPGC1α and at 48 h after infection, infected with Ad-ATF6α for 24 h. Western blot analysis show expression of ERRγ. Quantification of immunoblot is also shown. *P < 0.05, and **P < 0.005 using Student’s *t*-test. Data are representative of three independently performed experiments. (**C**) AML12 cells were infected with Ad-GFP, Ad-ERRγ or Ad-ERRγ along with Ad-PGC1α for 24 h, or first infected with Ad-shPGC1α and at 48 h after infection, infected with Ad-ERRγ for 24 h. Western blot analysis shows expression of ATF6α-N. Quantification of immunoblot is also shown. *P < 0.05 using Student’s *t*-test. Data are representative of three independently performed experiments. (**D**) AML12 cells were treated with DMSO or Tm (5 µg/ml) for 12 h followed by immunoprecipitation with the indicated antibodies showing physical interaction of PGC1α with ERRγ (left panel) and PGC1α with ATF6α (right panel). Data are representative of three independently performed experiments.(**E**) Co-localization of PGC1α with ERRγ (upper panel) and ATF6α (lower panel) in Hela cells. Data are representative of three independently performed experiments.

Next, to elucidate the role of PGC1α in this ATF6α-ERRγ cross talk in more detail, a transient transfection assay was performed with ERRγ and the ATF6α promoter (Figure 6A left and right panel, respectively). Tm treatment significantly increased both ERRγ and ATF6α promoter activity but this activation was severely compromised when endogenous PGC1α was knocked down. Next, Ad-ATF6α along with Ad-PGC1α significantly increased the ERRγ protein level but this protein level was significantly reduced on knockdown of endogenous PGC1α in AML12 cells (Figure 6B). Similar results were observed on overexpression of ERRγ and PGC1α in AML12 cells. Ad-ERRγ along with Ad-PGC1α significantly increased the ATF6α-N protein level but this protein level was significantly reduced on knockdown of endogenous PGC1α (Figure 6C). Next, we tested whether PGC1α physically interacts with ERRγ and ATF6α. In absence of ER stress, PGC1α interacted with both ERRγ and ATF6α, but this interaction was significantly enhanced for both ERRγ and ATF6α in presence of ER stress (Figure 6D left and right panel, respectively). To investigate whether PGC1α-ERRγ and PGC1α-ATF6α were co-localized in the same subcellular compartment, confocal microscopy was performed in HeLa cells (Figure 6E upper and lower panel). Our results demonstrated that both ERRγ and ATF6α co-localized with PGC1α in the nucleus as can be evidenced from the merged image. Overall we demonstrate that PGC1α is the key element in the cross talk between ERRγ and ATF6α.

### Dependence of both ERRγ and ATF6α on each other

**Figure 7.**
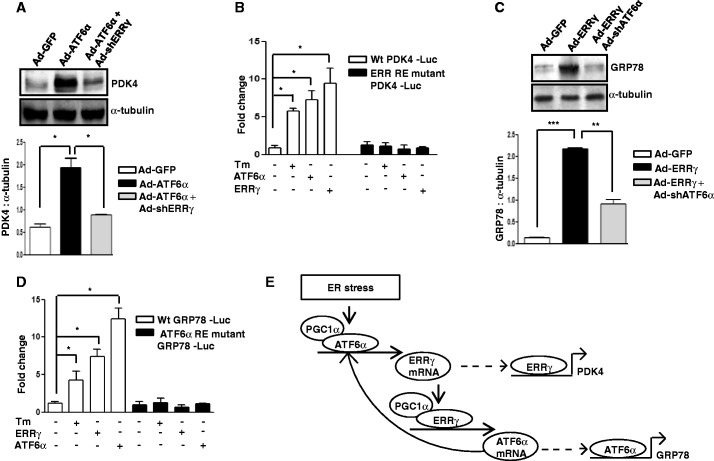
ATF6α and ERRγ reciprocally trans-activate each other. (**A** and **C**) Regulation of ERRγ target gene PDK4 by ATF6α (A) and regulation of ATF6α target gene GRP78 by ERRγ (C). (A) AML12 cells were infected with Ad-GFP or Ad-ATF6α for 24 h or first infected with Ad-shERRγ and at 48 h after infection, infected with Ad-ATF6α for 24 h. Western blot analysis shows expression of PDK4. Quantification of immunoblot is also shown. *P < 0.05 using Student’s *t*-test. Data are representative of three independently performed experiments, or (C) AML12 cells were infected with Ad-GFP or Ad-ERRγ for 24 h or first infected with Ad-shATF6α and at 48 h after infection, infected with Ad-ERRγ for 24 h. Western blot analysis shows expression of GRP78. Quantification of immunoblot is also shown. **P < 0.005, and ***P < 0.0005 using Student’s *t*-test. Data are representative of three independently performed experiments. (**B** and **D**) Activation of ERRγ target gene PDK4 promoter by ATF6α is mediated through ERRRE (B) and activation of ATF6α target gene GRP78 promoter by ERRγ is mediated through ATF6αRE (D). Transient transfection was performed in 293T cells with the indicated plasmid DNAs followed by Tm treatment. Data are representative of three independently performed experiments and shown as mean ± SD; *P < 0.05 using Student’s *t*-test. (**E**) Schematic representation of the proposed model in which ER stress–mediated induction of ATF6α and ERRγ depend on each other.

Finally, we examined the regulation of target genes of both ERRγ and ATF6α, as our results (Figures 2–5) demonstrated that ATF6α regulates ERRγ gene expression on ER stress and *vice versa*. As PDK4 is an ERRγ target gene (9), we examined whether ectopic expression of ATF6α had any role in PDK4 gene expression. Overexpression of ATF6α significantly increased PDK4 expression, and this increase was significantly attenuated on endogenous ERRγ knockdown in AML12 cells (Figure 7A). To provide insight into the mechanism, we mutated the ERRRE in the PDK4 gene promoter. Where both Tm and ATF6α activated the wild-type PDK4 promoter, on mutation of the ERRRE, neither Tm nor ATF6α could activate the PDK4 promoter (Figure 7B). Likewise, as GRP78 is a target gene of ATF6α (58), we analyzed whether ectopic expression of ERRγ had any regulatory role in GRP78 gene expression. Overexpression of Ad-ERRγ significantly increased GRP78 protein, but this increase was significantly compromised on knockdown of endogenous ATF6α by Ad-shATF6α in AML12 cells (Figure 7C). Mutation of the ATF6αRE on the GRP78 promoter destroyed activation by either Tm or ERRγ (Figure 7D). Taken together, we demonstrate that Tm/ATF6α can induce ERRγ target genes by regulating ERRγ itself and Tm/ERRγ can induce ATF6α target genes by regulating ATF6α gene expression.

## DISCUSSION

ER stress activates the unfolded protein response to generate multiple transcription factors that function in different cellular phenomena, including chromatin remodeling (35,45,10,11,59). In relation to coactivation of both ERRγ and ATF6α by ER stress, the present study provides direct evidence for ER stress in induction of ERRγ via ATF6α, but also for a newly recognized function of ERRγ in transcriptional regulation of ATF6α in response to ER stress. Moreover, physical and functional interactions of both ERRγ and ATF6α with coactivator provide a mechanistic basis for the ER stress–mediated induction of ERRγ and ATF6α gene expression and suggest the importance of chromatin remodeling during subsequent transcriptional events.

Our investigation of transcriptional cross talk between the nuclear receptor ERRγ and the ER-membrane–bound bZIP transcription factor ATF6α was motivated by findings that demonstrate many nuclear receptors either regulate specific transcription factors during ER stress or are regulated by them (41,35). Here, we investigated the role of ER stress in ERRγ gene expression and subsequently how ERRγ regulates ER stress–induced transcription factors. We observed a significant enhancement in ERRγ gene expression in response to the ER stressor Tm (Figure 1) that further establishes the interconnection between nuclear receptors and ER stress. Several previous studies show that X box binding protein 1 (XBP-1), which is similar to ATF6α, regulates ERα transcriptional activity through large-scale chromatin unfolding (37,38). Our findings suggest that ATF6α mediates the effect of ER stress on ERRγ (Figure 2A–D). These findings were further supported by a significantly low ERRγ protein level in ATF6α-null cells under basal conditions, as well as on Tm treatment. Previously, it was reported that ATF6α activates target genes through direct binding to an ATF6αRE in the promoters of target genes (17). This led us identify one ATF6α binding site in the ERRγ promoter. ChIP assay further confirmed ATF6α binding to the ERRγ promoter in response to ER stress. It has been reported that PGC1α is closely associated with the transcriptional activity of ATF6α (22). We also found that PGC1α plays an important role in ATF6α-mediated regulation of ERRγ gene expression. Using an *in vivo* ChIP assay we demonstrated significant recruitment of PGC1α to ATF6αREs in the ERRγ promoter during ER stress. Gene repression is often associated with decreased histone acetylation (56). Chromatin remodeling also occurs during coactivator gene transcription (60). In agreement with previous reports, we observed a significant increase in template-associated histone H3 and H4 acetylation at the ATF6αRE in the ERRγ promoter (Figure 3F). PDK4 is a member of the pyruvate dehydrogenase kinase superfamily (PDK1, −2, −3, −4) that regulates glucose metabolism. ERRγ induces PDK4 expression (61). Hypoxia and/or nutrient deprivation cause ER stress to induce PDK4 transcription through induction of ERRγ (9). ER stress potentiates hepatic insulin resistance (62), and insulin suppresses PDK4 expression (63). Therefore, during ER stress, defective insulin signaling might induce PDK4 expression. In accordance with these previous findings, we observed that Tm increased activity of the PDK4 promoter (Figure 7B). Moreover, overexpression of ATF6α significantly induced PDK4 expression, which was significantly attenuated by knockdown of endogenous ERRγ. The findings show that ATF6α induces ERRγ to activate ERRγ target genes (Figure 7A). Our results that link transactivation of ATF6α to ERRγ gene expression, along previous evidence supporting an interconnection between ER stress and nuclear receptor signaling (35,40), raise the possibility (discussed further below) that ATF6α may serve as a key mediator in ER stress–induced ERRγ gene expression.

CREBH, an ER-membrane–bound transcription factor that is similar to ATF6α in structure and mode of activation, is regulated by nuclear receptor PPARα (40), HNF4α (41) and GR (64). These findings along with a previous report (35) suggest a probable bidirectional regulatory pathway between transcription factors that regulate the mammalian ER stress response and nuclear receptors. To our surprise, overexpression of ERRγ significantly increased ATF6α gene expression. This result demonstrates a cross talk between ERRγ and ATF6α. In accordance with our observations (Figure 4A–C), the Tm-mediated increase in ATF6α protein was significantly decreased on either GSK5182 treatment or knockdown of endogenous ERRγ, supporting an apparent regulatory role of ERRγ for ATF6α expression during ER stress. It was previously reported that ERRγ binds to the sequence motif AGGTCA (17). A closer investigation of the ATF6α promoter revealed the existence of a probable ERRRE. Mutation of this site blocked promoter activation by Tm, demonstrating the importance of the ERRRE in ATF6α promoter function. Chip assay further confirmed evidence for ERRγ binding to the ATF6α promoter upon ER stress. PGC1α is closely associated with the transcriptional activity of ERRγ (3). Several lines of evidence indicate that transcriptional regulation of PDK4 expression by PGC1α is mediated by ERRα or ERRγ (61). In accordance with these previous reports, ER stress induced occupancy of PGC1α at the ERRRE in the ATF6α promoter, indicating PGC1α is a coactivator for ERRγ. Chromatin remodeling was also identified as a significant increase in the acetylation of histone H3 and H4 at the ATF6α promoter on Tm treatment or on ERRγ overexpression. The findings demonstrate chromatin remodeling during gene transcription as reported previously (57,60). BiP/GRP78 is a Ca^2+^-dependent ER chaperone that is induced by ER stress (21). Proteolytic cleavage and activation of ATF6α in response to ER stress upregulates GRP78 transcription (58). GRP78 participates in protein folding, transport and degradation upon ER stresses (65). A significant increase in GRP78 expression was observed upon ERRγ overexpression, and this increase was significantly attenuated by knockdown of endogenous ATF6α, suggesting that ERRγ induces ATF6α to activate transcription of the ATF6α target gene GRP78 (Figure 7C). Overall, our current findings reveal a novel molecular mechanism of reciprocal transcriptional activation used by ERRγ and ATF6α and provide evidence for a new role of both ERRγ and ATF6α in working in concert with PGC1α to affect chromatin remodeling in target genes.

Both ATF6α and ERRγ are implicated in numerous biological events. Previously, induction of liver steatosis and lipid droplet formation in ATF6α-knockout mice was reported (66,67). ATF6α is required for adipogenesis (68). ATF6α-null mice were reported to be glucose intolerant owing to pancreatic β-cell failure on a high-fat diet (69). The unfolded protein response mediates adaptation to exercise in skeletal muscle through a PGC-1α/ATF6α complex (22). ERRγ also plays critical role in cellular physiology. ERRγ modulates energy metabolism target genes in human trophoblasts (70). Previously our laboratory reported that ERRγ regulates hepatic gluconeogenesis (6) and LIPIN1, a gene involved in lipid metabolism (7). Glucosamine-induced ER stress causes insulin resistance in both human and rat skeletal muscle and impairs GLUT4 production and insulin-induced glucose uptake via an ATF6α-dependent decrease of the GLUT4 regulators MEF2A and PGC1α. Inhibition of ATF6α is sufficient to completely prevent glucosamine-induced inhibition of GLUT4, MEF2A and PGC1α in skeletal muscle cells (71). Moreover, viral infection induces ER stress, and hence induces both GRP78/BiP and ATF6α to facilitate protein folding during viral maturation (72). For example, the final assembly of rotavirus particles that cause severe diarrhea among infants and young children takes place in the ER. Protein disulfide isomerase, GRP78, calnexin and calreticulin are protein chaperones of the ER that are involved in the quality control of rotavirus morphogenesis. Cells with reduced expression of these chaperones exhibit defective maturation of rotavirus (73). All these chaperones are positively regulated at the transcriptional level by ATF6α (74,75). Therefore, inhibition of ATF6α might be an effective therapeutic target against rotavirus infection.

Here, we provide a previously unknown mechanism of regulatory pathway for ERRγ and ATF6α. We hypothesize that there are two parts of this regulatory loop (Figure 7E). In response to ER stress, the expression of both ERRγ and ATF6α increases, although ERRγ transcription is induced earlier than ATF6α (Figure 4B). On one hand, ATF6α along with coactivator mediates the increase in ERRγ transcription; on the other hand, ERRγ in association with coactivator mediates the increase in ATF6α transcription. Overall, our current findings provide insight into a novel chromatin remodeling strategy used by ERRγ and ATF6α. From a broader perspective, it may be relevant for different important biological processes in which both ERRγ and ATF6α play well-documented key regulatory roles.

## SUPPLEMENTARY DATA

Supplementary Data are available at NAR Online: Supplementary Table 1, Supplementary Figures 1–3 and Supplementary References [76–79].

## FUNDING

National Creative Research Initiatives Grant [2011-0018305] from the Korean Ministry of Education, Science, and Technology and the Future-based Technology Development Program (BIO Fields) through the National Research Foundation of Korea funded by the Ministry of Education, Science, and Technology Grant [20100019512 to H.S.C.], [2012M3A9C3048686 and 2011-0011433 to S.H.B] and the Korea Research Institute of Bioscience and Biotechnology (KRIBB) Research Initiative Program (to C.H.L.). Portions of this work were supported by National Institutes of Health (NIH) grants [DK042394, DK088227, HL057346R01, HL052173 and DK093074 to R.J.K.]. Funding for open access charge: National Creative Research Initiatives Grants from the Korean Ministry of Education, Science, and Technology and the Future-based Technology Development Program (BIO Fields) through the National Research Foundation of Korea funded by the Ministry of Education, Science, and Technology Grants, KRIBB Research Initiative Program grants, and NIH grants.

*Conflict of interest statement.* None declared.

## Supplementary Material

Supplementary Data
