# Supplementary material for: Transcriptional cross talk between orphan nuclear receptor ERRγ and transmembrane transcription factor ATF6α coordinates endoplasmic reticulum stress response
Source: Nucleic Acids Res. 2013 May 28;41(14):6960–74. doi: 10.1093/nar/gkt429 (PMC3737538; doi:10.1093/nar/gkt429)
Supplement: Supplementary Data [file supp_41_14_6960__index.html]

Transcriptional cross talk between orphan nuclear receptor ERRγ and transmembrane transcription factor ATF6α coordinates endoplasmic reticulum stress response — Transcriptional cross talk between orphan nuclear receptor ERRγ and transmembrane transcription factor ATF6α coordinates endoplasmic reticulum stress response — Supplementary Data 

# Transcriptional cross talk between orphan nuclear receptor ERRγ and transmembrane transcription factor ATF6α coordinates endoplasmic reticulum stress response

## Supplementary Data

files

**Files in this Data Supplement:**

- Supplementary Data - docx file
- Supplementary Data - pptx file
- Supplementary Data - pptx file
- Supplementary Data - pptx file
- Supplementary Data - pptx file
- Supplementary Data - docx file
- Supplementary Data - docx file
