# Supplementary material for: Transcriptional cross talk between orphan nuclear receptor ERRγ and transmembrane transcription factor ATF6α coordinates endoplasmic reticulum stress response
Source: Nucleic Acids Res. 2013 May 28;41(14):6960–74. doi: 10.1093/nar/gkt429 (PMC3737538; doi:10.1093/nar/gkt429)
Supplement: Supplementary Data [file supp_gkt429_nar-00443-v-2013-File009.docx]

Supplemental data statement

We have submitted three “supplemental figures”, one “supplemental table”, one “supplemental references list” and one “supplemental figure legends” file along with our “revised manuscript” and the “rebuttal letter”. Supplemental references (76-79) are also present in the main manuscript.
