# Supplementary material for: Transcriptional cross talk between orphan nuclear receptor ERRγ and transmembrane transcription factor ATF6α coordinates endoplasmic reticulum stress response
Source: Nucleic Acids Res. 2013 May 28;41(14):6960–74. doi: 10.1093/nar/gkt429 (PMC3737538; doi:10.1093/nar/gkt429)
Supplement: Supplementary Data [file supp_gkt429_nar-00443-v-2013-File010.pptx]

## Slide 1
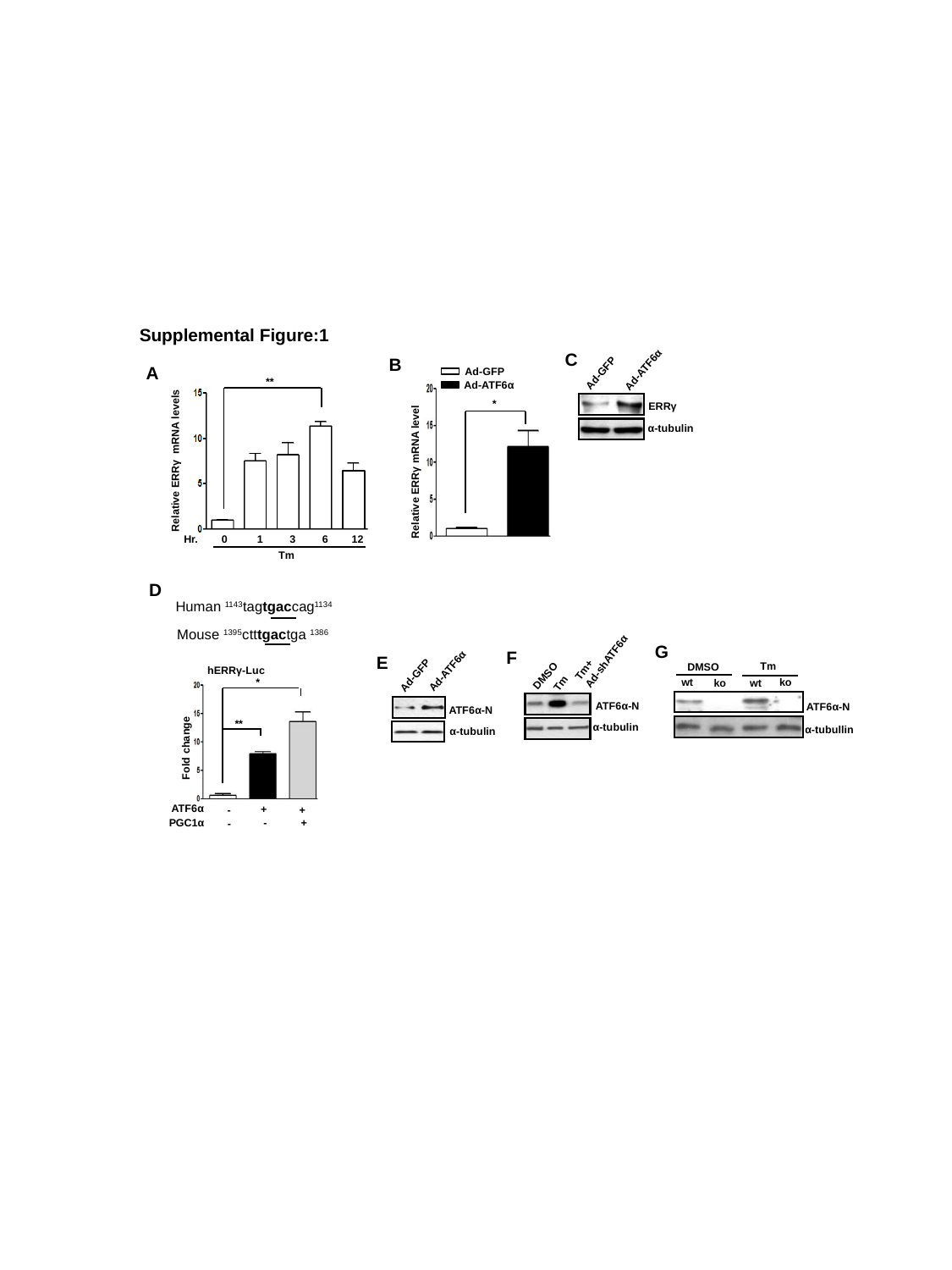

Supplemental Figure:1
C
Ad-GFP
ERRγ
α-tubulin
Ad-ATF6α
B
Ad-GFP
Ad-ATF6α
Relative ERRγ mRNA level
*
A
**
Relative ERRγ mRNA levels
Hr. 0 1 3 6 12
Tm
D
 hERRγ-Luc
Fold change
ATF6α
+
-
+
+
PGC1α
-
-
*
**
Human 1143tagtgaccag1134
Mouse 1395ctttgactga 1386
Tm+
Ad-shATF6α
F
DMSO
Tm
α-tubulin
ATF6α-N
Ad-ATF6α
Ad-GFP
ATF6α-N
α-tubulin
E
G
Tm
DMSO
wt
ko
wt
ko
ATF6α-N
α-tubullin
