# Supplementary material for: Transcriptional cross talk between orphan nuclear receptor ERRγ and transmembrane transcription factor ATF6α coordinates endoplasmic reticulum stress response
Source: Nucleic Acids Res. 2013 May 28;41(14):6960–74. doi: 10.1093/nar/gkt429 (PMC3737538; doi:10.1093/nar/gkt429)
Supplement: Supplementary Data [file supp_gkt429_nar-00443-v-2013-File011.pptx]

## Slide 1
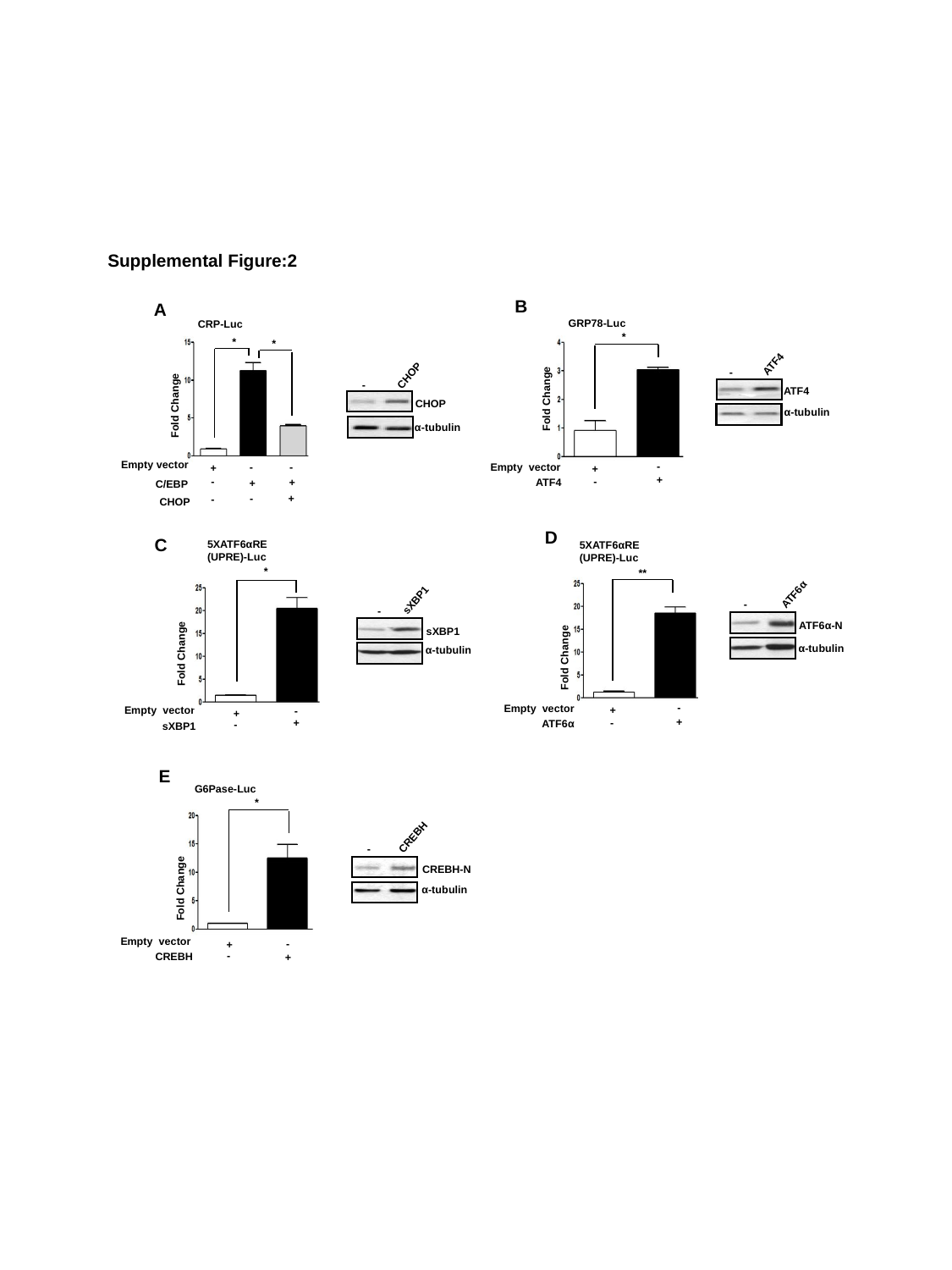

Supplemental Figure:2
B
Fold Change
Empty vector
GRP78-Luc
ATF4
*
-
+
+
-
A
CRP-Luc
Fold Change
Empty vector
C/EBP
CHOP
*
*
-
-
+
-
+
+
+
-
-
ATF4
α-tubulin
ATF4
-
CHOP
α-tubulin
CHOP
-
D
5XATF6αRE
(UPRE)-Luc
Fold Change
**
-
+
+
-
ATF6α
Empty vector
C
5XATF6αRE
(UPRE)-Luc
*
Fold Change
Empty vector
-
+
+
-
sXBP1
ATF6α-N
α-tubulin
ATF6α
-
sXBP1
α-tubulin
sXBP1
-
E
G6Pase-Luc
Fold Change
*
Empty vector
-
+
-
CREBH
+
CREBH-N
α-tubulin
CREBH
-
