# Supplementary material for: Transcriptional cross talk between orphan nuclear receptor ERRγ and transmembrane transcription factor ATF6α coordinates endoplasmic reticulum stress response
Source: Nucleic Acids Res. 2013 May 28;41(14):6960–74. doi: 10.1093/nar/gkt429 (PMC3737538; doi:10.1093/nar/gkt429)
Supplement: Supplementary Data [file supp_gkt429_nar-00443-v-2013-File012.pptx]

## Slide 1
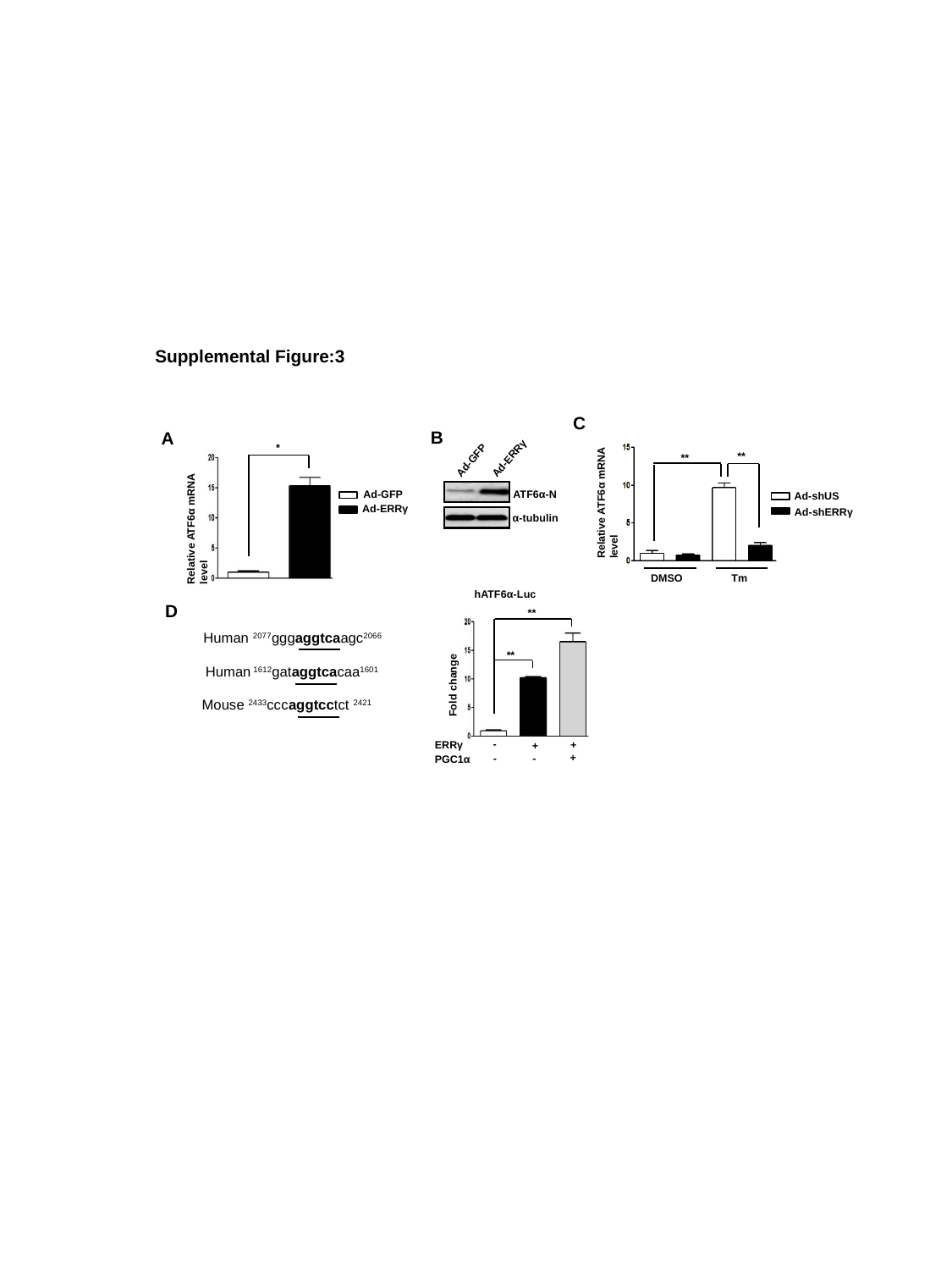

Supplemental Figure:3
B
Ad-GFP
ATF6α-N
α-tubulin
Ad-ERRγ
C
Relative ATF6α mRNA level
DMSO
Tm
Ad-shUS
Ad-shERRγ
**
**
A
Ad-GFP
Ad-ERRγ
Relative ATF6α mRNA level
*
 hATF6α-Luc
D
Fold change
-
ERRγ
+
+
+
-
-
PGC1α
**
**
Human 2077gggaggtcaagc2066
Human 1612gataggtcacaa1601
Mouse 2433cccaggtcctct 2421
