# Supplementary material for: Transcriptional cross talk between orphan nuclear receptor ERRγ and transmembrane transcription factor ATF6α coordinates endoplasmic reticulum stress response
Source: Nucleic Acids Res. 2013 May 28;41(14):6960–74. doi: 10.1093/nar/gkt429 (PMC3737538; doi:10.1093/nar/gkt429)
Supplement: Supplementary Data [file supp_gkt429_nar-00443-v-2013-File013.pptx]

## Slide 1
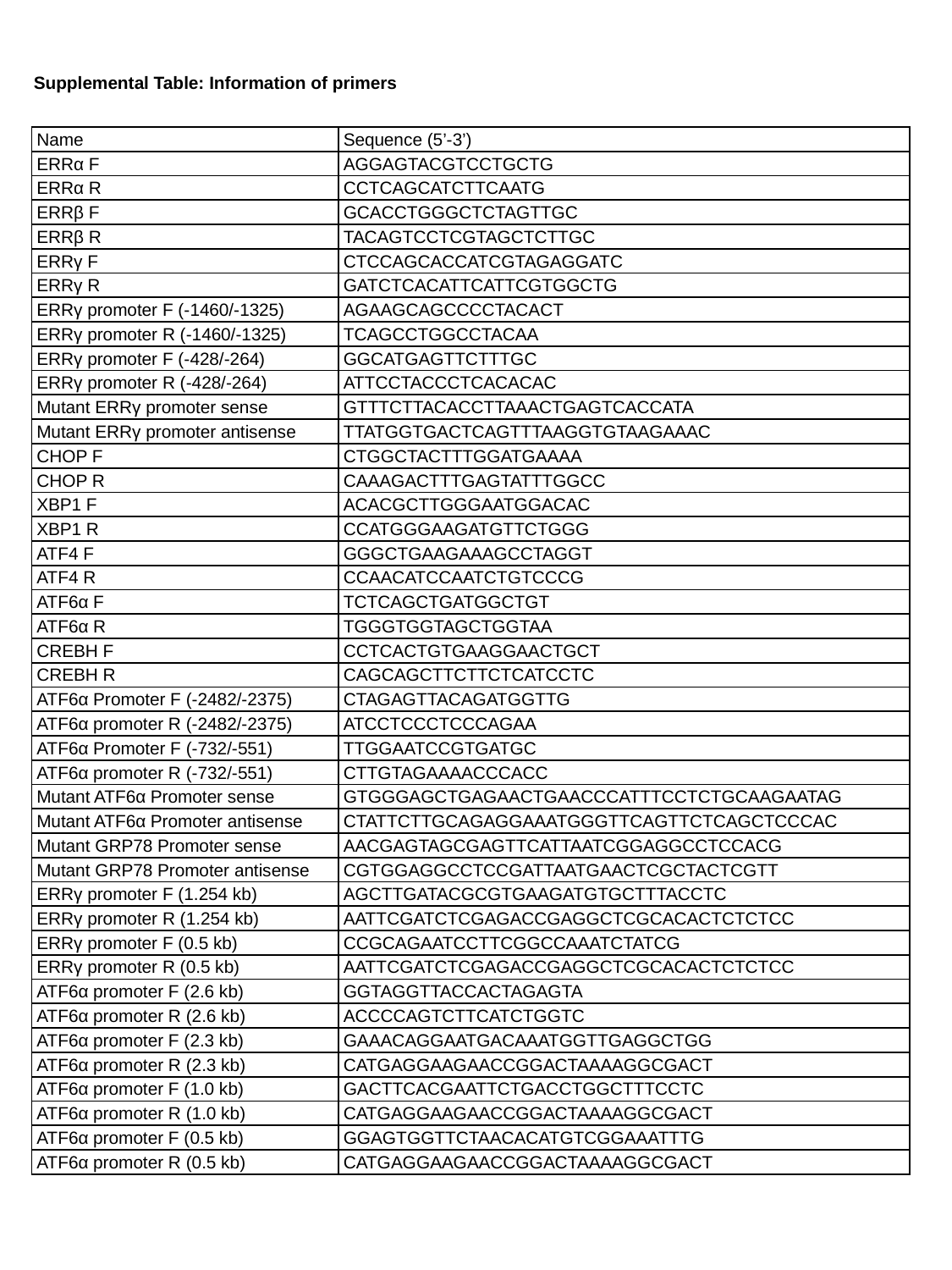

Supplemental Table: Information of primers
| Name | Sequence (5’-3’) |
| --- | --- |
| ERRα F | Aggagtacgtcctgctg |
| ERRα R | Cctcagcatcttcaatg |
| ERRβ F | GCACCTGGGCTCTAGTTGC |
| ERRβ R | TACAGTCCTCGTAGCTCTTGC |
| ERRγ F | Ctccagcaccatcgtagaggatc |
| ERRγ R | Gatctcacattcattcgtggctg |
| ERRγ promoter F (-1460/-1325) | Agaagcagcccctacact |
| ERRγ promoter R (-1460/-1325) | Tcagcctggcctacaa |
| ERRγ promoter F (-428/-264) | ggcatgagttctttgc |
| ERRγ promoter R (-428/-264) | attcctaccctcacacac |
| Mutant ERRγ promoter sense | Gtttcttacaccttaaactgagtcaccata |
| Mutant ERRγ promoter antisense | Ttatggtgactcagtttaaggtgtaagaaac |
| CHOP F | Ctggctactttggatgaaaa |
| CHOP R | Caaagactttgagtatttggcc |
| XBP1 F | Acacgcttgggaatggacac |
| XBP1 R | Ccatgggaagatgttctggg |
| ATF4 F | Gggctgaagaaagcctaggt |
| ATF4 R | Ccaacatccaatctgtcccg |
| ATF6α F | Tctcagctgatggctgt |
| ATF6α R | tgggtggtagctggtaa |
| CREBH F | cctcactgtgaaggaactgct |
| CREBH R | CAGCAGCTTCTTCTCATCCTC |
| ATF6α Promoter F (-2482/-2375) | CTAGAGTTACAGATGGTTG |
| ATF6α promoter R (-2482/-2375) | atcctccctcccagaa |
| ATF6α Promoter F (-732/-551) | TTGGAATCCGTGATGC |
| ATF6α promoter R (-732/-551) | cttgtagaaaacccacc |
| Mutant ATF6α Promoter sense | gtgggagctgagaactgaacccatttcctctgcaagaatag |
| Mutant ATF6α Promoter antisense | ctattcttgcagaggaaatgggttcagttctcagctcccac |
| Mutant GRP78 Promoter sense | aacgagtagcgagttcattaatcggaggcctccacg |
| Mutant GRP78 Promoter antisense | cgtggaggcctccgattaatgaactcgctactcgtt |
| ERRγ promoter F (1.254 kb) | agcttgatacgcgtGAAGATGTGCTTTACCTC |
| ERRγ promoter R (1.254 kb) | aattcgatctcgagaccgaggctcgcacactctctcc |
| ERRγ promoter F (0.5 kb) | CCGCAGAATCCTTCGGCCAAATCTATCG |
| ERRγ promoter R (0.5 kb) | aattcgatctcgagaccgaggctcgcacactctctcc |
| ATF6α promoter F (2.6 kb) | GGTAGGTTACCACTAGAGTA |
| ATF6α promoter R (2.6 kb) | accccagtcttcatctggtc |
| ATF6α promoter F (2.3 kb) | GAAACAGGAATGACAAATGGTTGAGGCTGG |
| ATF6α promoter R (2.3 kb) | CATGAGGAAGAACCGGACTAAAAGGCGACT |
| ATF6α promoter F (1.0 kb) | GACTTCACGAATTCTGACCTGGCTTTCCTC |
| ATF6α promoter R (1.0 kb) | CATGAGGAAGAACCGGACTAAAAGGCGACT |
| ATF6α promoter F (0.5 kb) | GGAGTGGTTCTAACACATGTCGGAAATTTG |
| ATF6α promoter R (0.5 kb) | CATGAGGAAGAACCGGACTAAAAGGCGACT |
