# Supplementary material for: Transcriptional cross talk between orphan nuclear receptor ERRγ and transmembrane transcription factor ATF6α coordinates endoplasmic reticulum stress response
Source: Nucleic Acids Res. 2013 May 28;41(14):6960–74. doi: 10.1093/nar/gkt429 (PMC3737538; doi:10.1093/nar/gkt429)
Supplement: Supplementary Data [file supp_gkt429_nar-00443-v-2013-File014.docx]

Supplemental references

# 76. [Voleti, B](http://www.ncbi.nlm.nih.gov/pubmed?term=Voleti%20B%5BAuthor%5D&cauthor=true&cauthor_uid=22750226)., [Hammond, D.J. Jr](http://www.ncbi.nlm.nih.gov/pubmed?term=Hammond%20DJ%20Jr%5BAuthor%5D&cauthor=true&cauthor_uid=22750226)., [Thirumalai, A](http://www.ncbi.nlm.nih.gov/pubmed?term=Thirumalai%20A%5BAuthor%5D&cauthor=true&cauthor_uid=22750226). and [Agrawal, A](http://www.ncbi.nlm.nih.gov/pubmed?term=Agrawal%20A%5BAuthor%5D&cauthor=true&cauthor_uid=22750226). (2012) Oct-1 acts as a transcriptional repressor on the C-reactive protein promoter. [Mol Immunol.](http://www.ncbi.nlm.nih.gov/pubmed/22750226), 52, 242-8.

# 77. Ron, D., and Habener, J. F. (1992) CHOP, a novel developmentally regulated nuclear protein that dimerizes with transcription factors C/EBP and LAP and functions as a dominant-negative inhibitor of gene transcription. Genes Dev., 6, 439–453.

# 78. [Luo, S](http://www.ncbi.nlm.nih.gov/pubmed?term=Luo%20S%5BAuthor%5D&cauthor=true&cauthor_uid=12871976)., [Baumeister, P](http://www.ncbi.nlm.nih.gov/pubmed?term=Baumeister%20P%5BAuthor%5D&cauthor=true&cauthor_uid=12871976)., [Yang, S](http://www.ncbi.nlm.nih.gov/pubmed?term=Yang%20S%5BAuthor%5D&cauthor=true&cauthor_uid=12871976)., [Abcouwer, S.F](http://www.ncbi.nlm.nih.gov/pubmed?term=Abcouwer%20SF%5BAuthor%5D&cauthor=true&cauthor_uid=12871976). and [Lee, A.S](http://www.ncbi.nlm.nih.gov/pubmed?term=Lee%20AS%5BAuthor%5D&cauthor=true&cauthor_uid=12871976). (2003) Induction of Grp78/BiP by translational block: activation of the Grp78 promoter by ATF4 through and upstream ATF/CRE site independent of the endoplasmic reticulum stress elements. [J Biol Chem.](http://www.ncbi.nlm.nih.gov/pubmed/?term=ACTIVATION+OF+THE+Grp78+PROMOTER+BY+ATF4+THROUGH+AN+UPSTREAM+ATF%2FCRE+SITE+INDEPENDENT+OF+THE+ENDOPLASMIC+RETICULUM+STRESS+ELEMENTS*), 278, 37375-85.

79. [Shen, J](http://www.ncbi.nlm.nih.gov/pubmed?term=Shen%20J%5BAuthor%5D&cauthor=true&cauthor_uid=15804611). and [Prywes, R](http://www.ncbi.nlm.nih.gov/pubmed?term=Prywes%20R%5BAuthor%5D&cauthor=true&cauthor_uid=15804611). (2005) ER stress signaling by regulated proteolysis of ATF6. [Methods.](http://www.ncbi.nlm.nih.gov/pubmed/15804611), 35, 382-9.
