# Supplementary material for: Transcriptional cross talk between orphan nuclear receptor ERRγ and transmembrane transcription factor ATF6α coordinates endoplasmic reticulum stress response
Source: Nucleic Acids Res. 2013 May 28;41(14):6960–74. doi: 10.1093/nar/gkt429 (PMC3737538; doi:10.1093/nar/gkt429)
Supplement: Supplementary Data [file supp_gkt429_nar-00443-v-2013-File015.docx]

Fig. 1. Transcriptional regulation of ERRγ by ATF6α in human hepatoma cell line (HepG2). (A), Tm (1mg/kg, i.p. in 1% DMSO/DW) was administered into C57BL/6J mice (n=5 per group) for up to 12 hr. Following completion of the experiments, mice were sacrificed, liver tissues were obtained and total RNA was isolated to perform qRT-PCR to quantify ERRγ mRNA levels using ERRγ primers. (B, C), HepG2 cells were infected with Ad-GFP or Ad-ATF6α for 24 h. Total RNA was isolated for qRT-PCR analysis to quantify ERRγ mRNA levels (B) or total protein was isolated for western blot analysis to show expression of ERRγ (C). (D), 293T cells were transfected with human ERRγ-Luc (200 ng) along with the indicated plasmid DNAs. Sequence of putative ATF6α binding site on human ERRγ promoter is also shown. (E), Effectiveness of overexpression of ATF6α in AML12 cells is presented. (F), Effectiveness of knockdown of endogenous ATF6α in AML12 cells is presented. (G), Expression of ATF6α in wild type and ATF6α-null hepatocyte cell line under basal and Tm treated condition is presented. Data from each panel is representative of three independent experiments and is presented as mean±SD; *, P<0.05, **, p<0.005 using Student’s t-test.

Fig. 2. Expression and associated activity of CHOP, sXBP1, ATF4, ATF6α, and CREBH plasmid DNAs. (A, left panel), 293T cells were transfected with CRP promoter containing reporter (200 ng) along with the indicated plasmid DNAs. (A, right panel), AML12 cells were transfected with CHOP plasmid DNAs. At 24 hr. after transfection cells were harvested for Western blot analysis using CHOP antibody. (B, left panel), 293T cells were transfected with GRP78 promoter containing reporter (200 ng) along with the indicated plasmid DNAs. (B, right panel), AML12 cells were transfected with ATF4 plasmid DNAs. At 24 hr. after transfection cells were harvested for Western blot analysis using ATF4 antibody. (C, left panel), 293T cells were transfected with 5XATF6αRE-Luc (200 ng) along with the indicated plasmid DNAs. (C, right panel), AML12 cells were transfected with XBP1 plasmid DNAs. At 24 hr. after transfection cells were harvested for Western blot analysis using XBP1 antibody. (D, left panel), 293T cells were transfected with 5XATF6αRE-Luc (200 ng) along with the indicated plasmid DNAs. (D, right panel), AML12 cells were transfected with ATF6α plasmid DNAs. At 24 hr. after transfection cells were harvested for Western blot analysis using ATF6α antibody. (E, left panel), 293T cells were transfected with G6Pase promoter containing reporter (200 ng) along with the indicated plasmid DNAs. (E, right panel), AML12 cells were transfected with CREBH plasmid DNAs. At 24 hr. after transfection cells were harvested for Western blot analysis using CREBH antibody. Data from each panel is representative of three independent experiments and is presented as mean±SD; *, P<0.05, **, p<0.005 using Student’s t-test.

Fig. 3. Transcriptional regulation of ATF6α by ERRγ in human hepatoma cell line (HepG2). (A), HepG2 cells were infected with Ad-GFP or Ad-ERRγ for 24 h. Total RNA was isolated for qRT-PCR analysis to quantify ATF6α mRNA levels. (B), HepG2 cells were infected with Ad-GFP or Ad-ERRγ for 24 h. Western blot analysis shows expression of ATF6α-N. (C), AML12 cells were infected with Ad-shUS or Ad-shERRγ. At 48 hr. after tinfection, cells were treated with DMSO or Tm (5µg/mL) for 12 hr. Total RNA was isolated for qRT-PCR analysis to quantify ATF6α mRNA level. (D, left panel), sequence of putative ERRγ binding site on human ATF6α promoter is shown. (D, right panel), 293T cells were transfected with human ATF6α promoter (200 ng) along with the indicated plasmid DNAs. Data from each panel is representative of three independent experiments and is presented as mean±SD; *, P<0.05, **, p<0.005 using Student’s t-test.
